# *Pseudomonas* Phage ZCPS1 Endolysin as a Potential Therapeutic Agent

**DOI:** 10.3390/v15020520

**Published:** 2023-02-13

**Authors:** Fatma Abdelrahman, Rutuja Gangakhedkar, Gokul Nair, Gamal El-Didamony, Ahmed Askora, Vikas Jain, Ayman El-Shibiny

**Affiliations:** 1 Center for Microbiology and Phage Therapy, Zewail City of Science and Technology, 6th of October City 12578, Egypt; 2 Microbiology and Molecular Biology Laboratory, Indian Institute of Science Education and Research, Bhopal 462066, India; 3 Department of Microbiology and Botany, Faculty of Science, Zagazig University, Zagazig 44519, Egypt; 4 Faculty of Environmental Agricultural Sciences, Arish University, Arish 45511, Egypt

**Keywords:** antibiotic resistance, bacteriophages, foodborne pathogens, phage-encoded enzymes, thermo-stable, antibacterial tool

## Abstract

The challenge of antibiotic resistance has gained much attention in recent years due to the rapid emergence of resistant bacteria infecting humans and risking industries. Thus, alternatives to antibiotics are being actively searched for. In this regard, bacteriophages and their enzymes, such as endolysins, are a very attractive alternative. Endolysins are the lytic enzymes, which are produced during the late phase of the lytic bacteriophage replication cycle to target the bacterial cell walls for progeny release. Here, we cloned, expressed, and purified LysZC1 endolysin from *Pseudomonas* phage ZCPS1. The structural alignment, molecular dynamic simulation, and CD studies suggested LysZC1 to be majorly helical, which is highly similar to various phage-encoded lysozymes with glycoside hydrolase activity. Our endpoint turbidity reduction assay displayed the lytic activity against various Gram-positive and Gram-negative pathogens. Although in synergism with EDTA, LysZC1 demonstrated significant activity against Gram-negative pathogens, it demonstrated the highest activity against *Bacillus cereus*. Moreover, LysZC1 was able to reduce the numbers of logarithmic-phase *B. cereus* by more than 2 log_10_ CFU/mL in 1 h and also acted on the stationary-phase culture. Remarkably, LysZC1 presented exceptional thermal stability, pH tolerance, and storage conditions, as it maintained the antibacterial activity against its host after nearly one year of storage at 4 °C and after being heated at temperatures as high as 100 °C for 10 min. Our data suggest that LysZC1 is a potential candidate as a therapeutic agent against bacterial infection and an antibacterial bio-control tool in food preservation technology.

## 1. Introduction

The imprudent use of antibiotics has resulted in the frequent emergence of multidrug-resistant bacteria (MDR), which makes the clinical treatment of infectious diseases more challenging [1,2]. This has made MDR one of the most serious threats to global public health [3,4]. *Bacillus cereus* is a Gram-positive, spore-forming bacterium widely distributed in the environment. It is an opportunistic foodborne pathogen that causes both local and systemic infections [5,6]. Since it is primarily present in the soil, it can be transmit to different foods [7]. *B. cereus* is one of the foremost common causes of food poisoning in food industry sectors, causing severe gastrointestinal disorders [8]. In addition to their usual gastrointestinal problems, *B. cereus* can cause ocular infections [9], and can lead to catheter-associated bloodstream infections [10]. It can produce two types of toxins: a diarrheal toxin and an emetic toxin, which are associated with its pathogenicity and toxicity in developing severe diseases [8]. Hence, the development of new antimicrobial agents for controlling, preventing, and treatment of *B. cereus* infections is required.

Bacteriophages (phages in short) are the viruses that naturally infect and propagate in their specific host. They, therefore, are the most promising alternative to antibiotics [11,12,13]. Bacteriophages have been exploited in various fields, such as the medical field, food, milk, yogurt, and brewing industries as well. In the food industry, bacteriophages have been used to eliminate microbes during primary production in poultry products controlling *Campylobacter* sp., swine products controlling *S. typhimurium*, and cattle products controlling *E. coli* [14,15,16]. Bren, 2007 has developed a phage-based product, ListShield^TM^ as a food additive to control *Listeria monocytogenes* in meat and poultry products [17]. Phages have also been used to control foodborne pathogens in post-harvest products such as meat carcasses, processed foods, powdered infant formula, fresh vegetables and fruits, and pasteurized milk [18].

In addition to using the whole phage particles for treating antibiotic-resistant bacteria, phage-encoded lytic enzymes also show great potential as candidate therapeutics for bacterial infections [19,20,21,22]. Phage lysins have many advantages over phages for being used as therapeutics, including non-proliferation, a broader host spectrum, easy-to-target drug delivery, and low bacterial resistance [23,24]. On the other hand, whole phages have much more complicated regulatory pathways and very low accessibility and bio-availability, as compared to the enzymes.

Phage lysozymes are already known to be used as an adduct in a few milk products, yogurt, and brewing industries and also as curative agents [25]. These enzymes are classified into three types based on their mechanism of action: (1) Endolysins which are produced in the host cytoplasm and are secreted after the phage infection and during the event of cell lysis to degrade the peptidoglycan, (2) Phage tail associated murein lytic enzymes (TAME) hydrolyze the peptidoglycan of the host cell post phage adsorption, allowing the injection of DNA inside the host [26,27], and (3) Polysaccharide depolymerases, which are responsible for EPS, CPS, and O-polysaccharide degradation. It can be virion-associated as an integral part of the virion particle or can be present in a soluble form which can be secreted during bacterial cell lysis [28].

Bacteriophage-encoded endolysins are further categorized into four classes based on their lytic activity: (1) N-acetylmuramoyl-L-alanine amidases (amidases), which cleave the amide bond between *N*-acetylmuramic acid (MurNAc) and L-alanine; (2) N-acetyl-β-D-muramidases (muramidases or lysozymes), which cleave β-1,4 glycosidic bonds between *N*-acetylmuramic acid (MurNAc) and *N*-acetyl-D-glucosamine (GlcNAc) residues; (3) endo-β-*N*-acetylglucosaminidases (glucosaminidases), which acts to catalyze the hydrolysis of the glycosidic bond of peptidoglycan; (4) endopeptidases, which cleave the peptide bond between two amino acids of the stem peptide [29,30].

In this study, we have identified a novel *Pseudomonas* phage ZCPS1 and carried out structural and functional characterization of its putative endolysin LysZC1. The enzyme shows remarkable antibacterial activity against various Gram-negative pathogens, when administered in combination with EDTA but demonstrates its highest activity against a serious food pathogen, *Bacillus cereus*. Interestingly, we found that LysZC1 is stable and active at different temperatures ranging from 10–100 °C, and after prolonged storage at 4 °C. Therefore, our study provides a potential and thermo-stable antibacterial agent against *B. cereus* and a solution for its contamination in food industries. We believe that the thermostable property of this enzyme can be exploited to engineer more efficient artilysins.

## 2. Materials and Methods

### 2.1. Bacterial Strains and Growth Conditions

*Escherichia coli* DH5α and BL21 (DE3) were used for cloning and protein production, respectively. Antimicrobial activity assays were carried out using *B. cereus* (ATCC 11778; American Type Culture Collection, Manassas, VA, USA), *B. cereus* (MTCC 1306; Microbial Type Culture Collection and Gene Bank, Chandigarh, India). *Staphylococcus aureus* MTCC 1430^T^. *Salmonella enterica* subsp. *enterica* serovar Typhimurium ATCC 14028, *Pseudomonas aeruginosa*, *E. coli* ATCC 8739, *Shigella sonnei* ATCC 29930, and *Klebsiella pneumoniae* MTCC 432 were used for turbidity reduction assay of Gram-negative pathogens. *B. cereus* MTCC 1306, *S. aureus* MTCC 1430T, and *K. pneumoniae* MTCC 432 were grown in Luria-Bertani (LB; Difco, Detroit, MI, USA), aerobically at 37 °C with constant shaking at 200 rpm whereas the rest of the bacterial strains were grown in Tryptic Soya Broth (TSB; Oxoid, Hampshire, UK), aerobically at 37 °C with constant shaking at 120 rpm. The stocks of bacterial strains were kept in 20% glycerol at −80 °C until needed.

### 2.2. In Silico Analysis

ZCPS1 phage genome was annotated using the myRAST algorithm [31] and PHASTER, and the alternative start codons were manually evaluated. The functional domain analysis of phage ZCPS1 and LysZC1 was performed by BLASTP search at NCBI [32]. LysZC1 properties, including isoelectric point (pI) and molecular weight were predicted using the ExPASY Prot Param tool.

### 2.3. Structure Prediction and Verification

LysZC1 protein structure was predicted using the Phyre2: Protein Fold Recognition Server [33]. Structural refinement and validation of predicted models were done using the SAVESv6.0 server. The protein model with the best score was selected for further analysis. Structural alignment was carried out using the PyMOL alignment module, while TM was calculated using the Tm score [34].

### 2.4. Molecular Dynamics (MD) Simulation Studies

The verified structure was subjected to the solution builder of the CHARMM-GUI web server in order to generate input files for molecular dynamics simulation of protein in an aqueous solvent environment [35]. The protein was hydrated using a water box with an edge distance of 10 Å, and 0.15 M NaCl was placed with the Monte-Carlo method to neutralize the charges. The system contained one protein molecule embedded in a water box along with 33 Na^+^ and 34 Cl^−^ ions. A CHARMM-36m force field and NPT (constant particle number, pressure, and temperature) ensemble with no external surface tension was used to generate the input files. MD simulation was carried out for 100 ns using GROMACS v2020.5 [36]. Stepwise energy minimization and equilibration were carried out and in the end; a production run was executed to generate the final trajectory. Analysis of the trajectory was carried out using GROMACS v2020.5 and visualized using PyMOL.

### 2.5. Construction of LysZC1 Expression Vector

LysZC1 encoding gene was PCR amplified (forward primer: 5′-GGCCATGGGCATGAAGTTCGACGAATACTC and reverse primer: 5′-CCTCGAGAGTCACCGTGCTACCTATC), digested with NcoI/XhoI restriction enzymes, and ligated with T4 ligase (NEB) into the same restriction sites in pET28b (Novagen, WI, USA), resulting in pET28b-LysZC1 encoding a lysin with a C-terminal hexa-histidine tag. The clone was confirmed by Sanger sequencing (Eurofins, Germany). The sequence of LysZC1 lysin was deposited in GenBank under accession no. UPO63074.

### 2.6. Recombinant Protein Production, and Purification

*E. coli* BL21(DE3) cells (NEB, MA, USA) transformed with pET28b-lysZC1 vector were grown in Luria Bertani (LB) broth (Neogen, MI, USA) supplemented with 50 µg/mL kanamycin at 37 °C with constant shaking at 150 rpm. The culture was induced with isopropyl-β-D-1-thiogalactopyranoside (IPTG) to a final concentration of 0.5 mM, after it reached an optical density at 600 nm (OD_600_) ~0.6 and was further incubated at 18 °C with constant shaking at 150 rpm overnight. Cells were then harvested by centrifugation (4000× *g*, 10 min, 4 °C), and the pellet was resuspended in lysis buffer (25 mM Tris-HCl pH 7.5, 150 mM NaCl, 5 mM imidazole, 5 mM MgCl_2_, 0.1% Triton X-100) supplemented with Protease Inhibitor Cocktail, EDTA -free (Sigma-Aldrich, St. Louis, MO, USA). Afterwards, cell disruption was achieved by three freeze-thaw cycles (from −80 °C to room temperature) followed by ultra-sonication on ice (Branson SFX150 Ultrasonic Processor, Mexico) for 10 cycles (30 s pulse, 30 s pause) at 40% amplitude. Insoluble cell debris was removed by centrifugation (10,000× *g*, 30 min, 4 °C), and the supernatant was filtered through a 0.22 μm filter membrane and mixed with 2 mL of Ni-NTA resin (GE Healthcare, Sweden) previously equilibrated with lysis buffer containing 5 mM imidazole. The protein-resin mixture was then loaded onto a polypropylene column (GE Healthcare), and the flow-through was discarded. The column was further washed with wash buffer 1 (40 mM Tris-HCl pH 7.8, 1 M NaCl, 20 mM imidazole, 5% glycerol, 5 mM β-mercaptoethanol, 0.1% Triton X-100) followed by wash buffer 2 (40 mM Tris-HCl pH 7.8, 0.5 M NaCl, 20 mM imidazole, 5% glycerol, 5 mM β-mercaptoethanol, 0.1% Triton X-100). Finally, LysZC1-6xHis was eluted with elution buffer (40 mM Tris-HCl pH 7.8, 0.5 M NaCl, 250 mM imidazole, 5% glycerol, 5 mM β-mercaptoethanol, 0.1% Triton X-100). Purified LysZC1-6xHis protein was analyzed on 15% SDS-PAGE gel electrophoresis. The gel was stained with Coomassie brilliant blue R-250 and imaged. The protein was buffer-exchanged against 25 mM Tris-HCl pH 7.5, 150 mM NaCl, and 5% glycerol using a PD10 column (GE Healthcare, UK) and stored at 4 °C.

### 2.7. Biophysical Analysis of the Proteins Using Circular Dichroism (CD) Spectroscopy

The proteins were dialyzed against CD buffer containing 20 mM Tris-Cl pH 8.0, 50 mM NaCl and 1 mM DTT, and 0.2 µg/µL of protein was subjected to CD spectroscopic analysis on a JASCO J-815 spectropolarimeter. Spectra were further processed using Spectra Manager software. Far-UV CD spectra were recorded at 20 °C in a 0.2 cm path length quartz cell. A total of three scans were obtained at a scanning speed of 100 nm/min, and data were averaged and blank subtracted. The protein was also analyzed for thermal stability by scanning for thermal melting from 5 °C to 95 °C and in reverse order and monitoring at 222 nm; the data were used for the Tm analysis. The data were blank subtracted and plotted.

### 2.8. Expression Analysis by Western Blotting

Western blotting was used to confirm the presence of the expected LysZC1 protein in the eluate. SDS-PAGE was used to resolve the protein samples, then transferred to a polyvinylidene fluoride (PVDF) membrane (Millipore, MA, USA) using the semi-dry transfer (turbo, Bio-Rad, CA, USA). After blocking the membrane for 1 h with 5% (*w*/*v*) skimmed milk in PBS (milk/PBS), it was incubated overnight at 4 °C with a 1:1000 dilution of primary antibody; mouse serum-containing anti-6xHis antibody (Thermo Fisher Scientific, MA, USA). Following three washes with Tris-buffered saline containing 0.1% Tween 20, the membrane was incubated with a 1: 10,000 dilution of goat anti-mouse horseradish peroxidase (HRP) secondary antibody (Jackson ImmunoResearch Laboratories, Inc.; PA, USA) for 1 h at room temperature. Following three washes with TBST, the blot was incubated in a 1:1 ratio with western bright ECL solutions (Thermo Scientific, IL, USA). ChemiDoc MP Imaging System (Bio-Rad, CA, USA) was used to capture the ECL signal.

### 2.9. Zymography

The activity of the purified LysZC1 was tested using 0.2% lyophilized *Micrococcus lysodeikticus* cells (Sigma-Aldrich, St. Louis, MO, USA) as substrate in a 12% SDS-polyacrylamide gel. 20 µg protein was boiled in a SDS sample loading buffer and loaded on the SDS-polyacrylamide gel containing the substrate. After electrophoresis, the gel was washed twice for 1 h with renaturation buffer containing 25 mM Tris-Cl, pH 8.0, and 1% Triton X-100, and incubated in the same buffer at 25 °C for 12 h followed by staining with 1% methylene blue in 0.01% KOH for 1 h and further de-staining with distilled water. The control SDS-PAGE gel was stained with Coomassie Brilliant Blue and de-stained further. BSA and lysozyme were used as negative and positive controls, respectively. The lytic activity of protein bands was observed as a clear zone against a blue background after washing the gel in ultrapure water.

### 2.10. Estimation of Minimum Inhibitory Concentration of LysZC1

The Minimum Inhibitory Concentration (MIC)of LysZC1 was determined by treating *Bacillus cereus* cells with OD_600_ = 1 with different concentrations of the enzyme (20, 40, 100, 200, and 400 µg/mL) and kinetically monitoring the reduction in the turbidity of the culture in terms of OD_600_ decrease, which is also termed as turbidity reduction assay. A 250 µL reaction was prepared with 200 µL of the substrate and 50 µL of the enzyme mixture containing enzyme and reaction buffer (40 mM Tris-HCl, pH 8; 200 mM NaCl, and 1 mM DTT). The OD decrease was monitored every 5 min, with intermittent shaking, for 2 h at 37 °C in a 96 well plate on SPECTRAmax PLUS (Molecular Devices, Sunnyvale, CA, USA) and analyzed using SoftMax Pro 5.4.1. LB media was used as blank and Lysozyme and BSA at 200 µg/mL were used as positive and negative controls, respectively. The assay was performed in triplicates, and the data is presented as the mean ± standard deviation (SD).

### 2.11. Antimicrobial Activity Assay of LysZC1

The Antimicrobial activity of LysZC1 was determined by a Turbidity reduction assay (as described in Section 2.10) and Colony-Forming Unit (CFU) reduction assay as previously described [37] with some modifications. Bacterial reference strain, *B. cereus* ATCC 11778, was cultured in TSB broth at 37 °C with shaking at 120 rpm for both the logarithmic (OD_600_ = 0.6–0.8) and stationary phase (OD_600_ = 1.4–1.6). The bacterial culture was then centrifuged at 4000× *g* at 4 °C for 10 min, and bacterial cells were washed twice in 20 mM Tris-HCl (pH 7.4) and then resuspended in the same buffer. The bacterial suspension was then treated with Lysin (LysZC1) at a final concentration of 100 μg/mL and incubated aerobically for 1 h at 37 °C. For negative control, bacterial suspension was treated with 20 mM Tris-HCl buffer instead of LysZC1. Afterward, the mixture was serially diluted and plated on the Typtic Soya Agar (TSA; Oxoid, UK) plates. The viable cell count was determined after the incubation overnight at 37 °C. We also monitored the effect of LysZC1 on logarithmic and stationary phage kinetically, as a time course assay exactly as described previously in Section 2.10. Lysozyme and BSA were used as positive and negative control. All assays were performed in triplicate, and the data were presented as the mean ± standard deviation (SD).

### 2.12. The Synergistic Antimicrobial Activity Assay of LysZC1 and EDTA

Antimicrobial activity against some Gram-negative bacterial strains was performed as described previously [38] with some modification. Briefly, *E. coli*, *S. typhimurium*, *S. sonnei*, and *P. aeruginosa* were cultured in TSB at 37 °C to the log phase of growth and *K. pneumoniae* was grown in LB broth at 37 °C till the log phase. Bacterial cultures were harvested at 4000× *g* at 4 °C for 10 min and then washed twice in 20 mM Tris-HCl, pH 7.4. The cell pellet was then resuspended in the same buffer and the OD_600_ was adjusted to 1.0. Bacterial suspensions were then treated with LysZC1 at a final concentration of 100 μg/mL with or without EDTA at a final concentration of 3 mM; in addition, a group treated with 3 mM EDTA only was taken as control. For negative controls, bacterial suspension was treated with 20 mM Tris-HCl buffer. The OD_600_ was measured over time using a microplate reader (FLUOstar Omega, BMG LABTECH, Ortenberg, Germany). The relative reduction in OD_600_ was calculated after 60 min as follows: Relative reduction in OD_600_ (%) = 100 × ΔOD_600_ of the treated sample/ the initial OD_600_ of control. All experiments were performed in triplicates.

### 2.13. Assessment of LysZC1 Lytic Activity at Different Temperatures and pH, upon Prolonged Storage

In order to check the effect of temperature on the activity of the protein, we performed the experiment at 10, 25, 45, 60, 80, and 100 °C. Till 45 °C, we maintained the reaction temperature and monitored the reduction in the turbidity of the culture, whereas, for 60–100 °C, we heated the protein at respective temperatures for 10 min, allowed it to cool, and then separated the precipitated protein by high-speed centrifugation at 13,400 rpm for 1 h. The soluble protein was quantified, and 100 μg/mL of LysZC1 protein was tested for activity at 37 °C. Lysozyme and BSA were used as positive and negative controls, respectively. A non-heated LysZC1 at 100 μg/mL was also used as a positive control in case of treatment at 60, 80, and 100 °C. We also verified the same result by spotting 5 µL of the protein with a final concentration of 100 μg/mL on *B. cereus* lawn on the TSA plate.

The activity of LysZC1 over different pH (4, 5, 6, 7, 8, 9, 10, and 11) was determined by washing *B. cereus* culture of OD_600_~1 with buffers of different pH (20 mM Sodium acetate for pH 4–5; 20 mM Tris Buffer for pH 6–8; 20 mM glycine for pH 9–10; and 20 mM sodium carbonate for pH 11) and re-suspended in the same buffer. The culture was further treated with 100 μg/mL of LysZC1 for 2 h at 37 °C. 

Similarly, the lytic activity was assessed at various time points against *B. cereus* by the spot inoculation method as previously described [36] with some modification. Purified LysZC1 was kept at 4 °C for 11 months. *B. cereus* was cultured in TSB broth and incubated at 37 °C until it reached the logarithmic phase (OD_600_ = 0.6–0.8). Then, the *B. cereus* culture was spread onto TSA plates. Subsequently, 5 µL of LysZC1 was spotted on the surface of the agar plates. The sensitivity to LysZC1 was observed by the formation of a clear lytic zone upon overnight incubation at 37 °C.

### 2.14. Scanning Electron Microscopy (SEM)

SEM was used to visualize the potential effect of LysZC1 on bacterial cells. The reference bacterial strain, *B. cereus*, at the logarithmic phase was centrifuged, washed, and resuspended in 20 mM Tris-HCL (pH 7.4). 800 μL of bacterial suspension were mixed with 200 μL of LysZC1 (500 μg/mL) or Tris-buffer as a control. After 2 h of incubation at 37 °C, bacterial cells were centrifuged at 4000× *g* for 10 min and washed twice with 20 mM Tris-HCl (pH 7.4), followed by fixation with 2.5 % (*v*/*v*) glutaraldehyde and washing with PBS. Samples were dehydrated in a series of ethanol (30, 50, 70, 80, 90 % (*v*/*v*), and absolute), coated with gold using JEOL JFC-100 sputter, and the morphology of the aggregated bacterial pellets was examined by SEM (JEOL JSM-5300, UK).

## 3. Results

### 3.1. The Lytic Pseudomonas Phage ZCPS1 Encodes a Putative Lysozyme LysZC1

We analyzed the genome of *Pseudomonas* phage ZCPS1 (GenBank ID: ON156559.1) and identified a putative lysozyme LysZC1, revealed by the BLAST search carried out with the query sequence against the non-redundant protein sequence database available at the NCBI. LysZC1 shows a high identity with the available *Pseudomonas* phages and other phage lysozyme sequences (Figure S1). Additionally, the conserved domain database search available at the NCBI server also demonstrated the presence of a lysozyme-like domain in it. Therefore, we conclude that LysZC1 is a lysozyme encoded by *Pseudomonas* phage ZCPS1, and codes for a 165 amino acid protein with a predicted molecular weight of 18,870 Da and an isoelectric point of 8.43.

**Figure 1:**
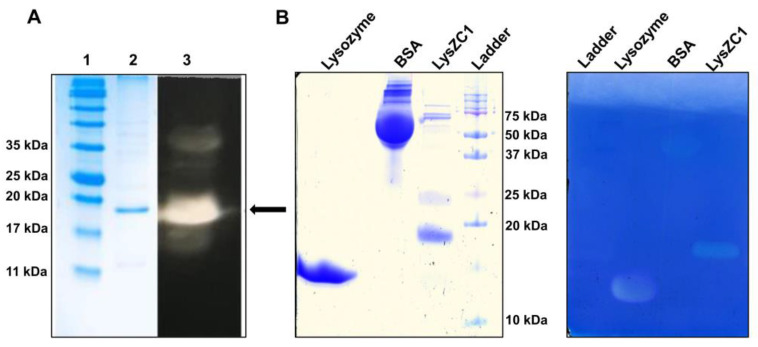
LysZC1 protein functions as a lysozyme. (**A**) SDS-PAGE and western blotting analysis for LysZC1. Lane 1, protein marker; Lane 2, Purified Lysin LysZC1 loaded on an SDS-PAGE gel (15%). Lane 3, Western blotting for LysZC1 protein expression; (**B**) Zymography of the LysZC1 protein. The left panel shows SDS-PAGE of various proteins used for zymography and right panel shows zymography of the same proteins against *Micrococcus lysodeikticus* with their respective zone of clearance. The zone of clearance due to the activity of the enzyme (LysZC1) is clearly visible. Lysozyme and BSA were used as positive and negative controls, respectively.

In order to establish experimentally that LysZC1 is indeed a functional lysozyme that can break down peptidoglycan and kill bacteria, we first cloned, expressed, and purified this protein from *E. coli* cells. The SDS-PAGE profile of the purified protein showed a single band corresponding to the molecular weight of recombinant lysin LysZC1 (~20 kDa; Figure 1A). Then, we examined the protein for its function. Although phage ZCPS1 infects and kills *Pseudomonas* sp., a Gram-negative bacterium, and therefore, its lysozyme is primarily meant for the disruption of *Pseudomonas* cell wall peptidoglycan, we carried out the functional assay of the purified LysZC1 using *Micrococcus lysodeikticus* cells as a substrate on a zymography, as described previously [39]. *M. lysodeikticus* is a Gram-positive organism that has its cell wall peptidoglycan as the outer layer of the cell envelope. We obtained a distinct zone of clearance on the Methylene Blue-stained SDS-PAGE gel (Figure 1B), which immediately suggests that the LysZC1 is a broad-spectrum lysozyme encoded by the phage ZCPS1. This broad spectral activity is likely due to the muramidase activity of the protein, which targets 1,4-beta-linkages between N-acetylmuramic acid and N-acetyl-D-glucosamine residues of peptidoglycan.

### 3.2. Structural Analysis of LysZC1 Indicates Similarity with Lysozyme-like Proteins

In order to characterize the LysZC1, we have carried out a BLAST search using LysZC1 amino acid sequence. The data suggests LysZC1 has high sequence similarity (60–90%) with the lysozyme of *Pseudomonas* phages while it also shares lesser but significant sequence similarity with glycoside hydrolase family protein from a wide range of phages (Table S1). Interestingly the multiple sequence alignment with the same set of hits suggests a high level of conservation (Figure S1). The highly conserved profile of LysZC1 with known bacteriophage lysozymes generates a strong possibility that LysZC1 is a lysozyme-like protein.

Since the crystal structures of any high sequence similarity proteins are unavailable, we constructed a 3D model of LysZC1 using Phyre2 Protein Fold Recognition Server [33]. [The server predicted the structure of LysZC1 protein using the muramidase domain of spmx from *Asticaccaulis excentricus* as a template which has 34.52% identity with the LysZC1 sequence.

**Figure 2:**
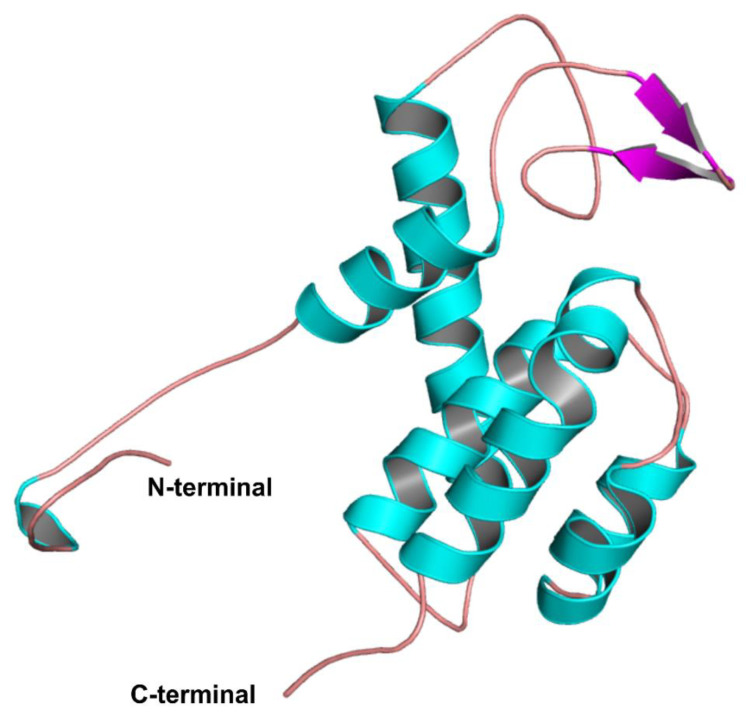
MD simulation of LysZC1 protein. The protein structure post MD simulation was visualized with PyMOL. Alpha helix, beta-sheet and loops are shown with cyan, magenta and salmon red colour respectively.

The generated model was further validated using the SAVESv6.0 server. The validated structure was then subjected to 100 ns MD simulation using GROMACS v2020.5, which finally yielded a stable structure. The final 3D model of LysZC1 consists of six helices—α1 (L22-E32), α2 (L62-Q83), α3 (P94-L107), α4 (S120-A124), α5 (F127-W132), α6 (P144-A157) along with β-sheet (Y39-D41), (Y45-T47) and loop regions (Figure 2).

**Figure 3:**
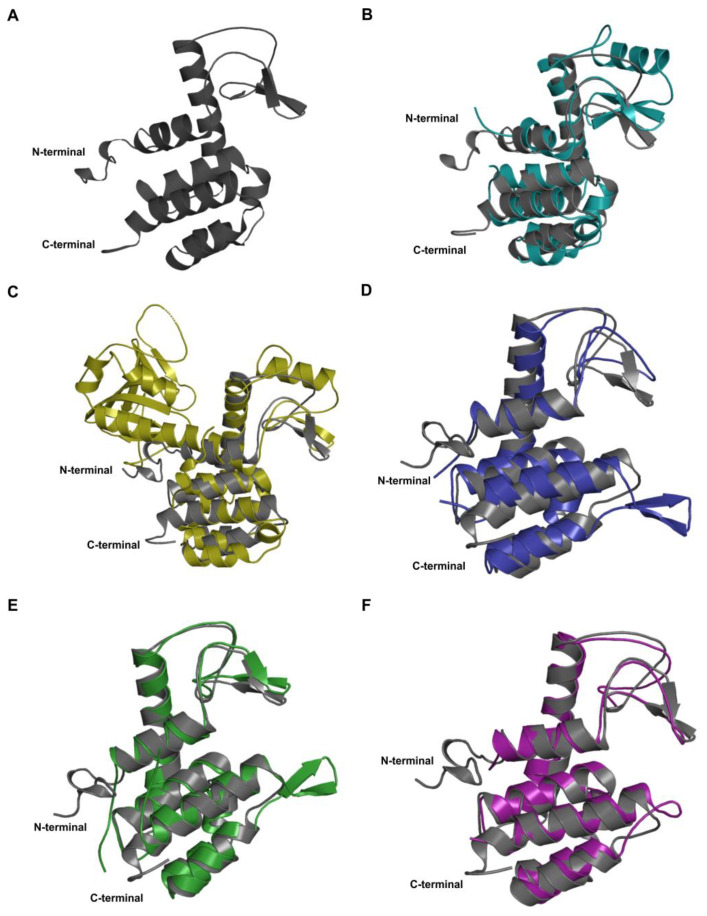
Structural analysis of LysZC1 protein. (**A**) Phyre2 server generated LysZC1 protein structure (grey colour). Structural alignment of predicted LysZC1 protein structure with (**B**) Lysozyme 056 from Deep neural language modeling (deep teal colour), (**C**) Crystal structure of a pesticin and T4-lysozyme chimera (olive colour), (**D**) Muramidase domain of SpmX from *Asticaccaulis excentricus* (deep blue colour), (**E**) Endolysin from *Escherichia coli* O157:H7 phage FAHEc1 (forest green colour), (**F**) Crystal structure of muramidase from *Acinetobacter baumannii* AB 5075UW prophage (deep purple colour).

Structural alignment of the predicted structure was performed with few available phage lysin structures having significant sequence identity with LysZC1. Data suggests significant structural alignment, especially in helices. (Figure 3) The TM score suggests that the predicted structure is in about the same fold of the templates and not any random structural similarity (Table S2). Altogether structural alignment data indicates all the structure exhibits significant structural homology. Despite the moderate sequence similarity between LysZC1 and the templates used for structural alignment, the aligned region showed a high level of conservation, thus indicating a high possibility of functional similarity (Figure S2). All the structural and sequence analysis data, along with the ability of LysZC1 protein to hydrolyze peptidoglycan, clearly indicate the muramidase nature of the protein.

### 3.3. Biophysical Analysis Shows LysZC1 to Be a Well-Folded and Reasonably Stable Protein

**Figure 4:**
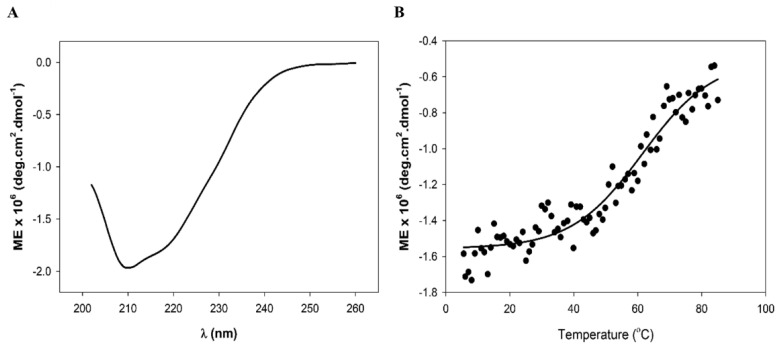
Circular Dichroism spectroscopic analysis of LysZC1. (**A**) Plot shows the CD spectrum of LysZC1. (**B**) Thermal stability analysis of the protein at indicated temperatures. Only 222 nm data are plotted. In both panels (**A**,**B**), the data are plotted as molar ellipticity.

We further carried out the circular dichroism analysis of LysZC1 to verify our in-silico observation presented above. The purified protein was subjected to CD analysis on a JASCO J815 spectropolarimeter. The profile obtained (Figure 4A) was further analyzed for the secondary structure content using the K2D2 webserver. Our data suggest that the protein consists of both alpha helix and beta sheet along with random coils, with a prominent helical content. This validates our in-silico data, which shows more helices than the beta sheet in the protein. We next measured the thermal stability of the protein. We allowed the protein to denature by increasing the temperature from 5 °C to 95 °C. The T_m_ of the protein was found to be 61.4 °C (Figure 4B).

### 3.4. LysZC1 Shows Highest Bactericidal Activity against Bacillus cereus

**Figure 5:**
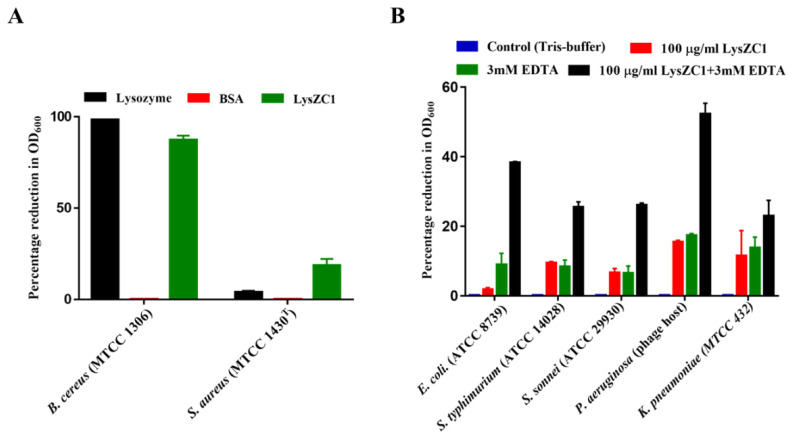
Activity of LysZC1 on Gram-positive and Gram-negative foodborne pathogens. (**A**) Turbidity reduction assay of LysZC1 against *Bacillus cereus* and *Staphylococcus aureus* (OD_600_ = 1). Lysozyme and BSA were used as controls. (**B**) The plot shows the lytic activity of 100 µg/mL LysZC1 with and without 3 mM EDTA on different Gram-negative bacterial strains as indicated. Tris-buffer was used as negative control. Data are shown as Mean ± SD.

We carried out the turbidity reduction assay to determine the lytic activity of LysZC1 against commonly known Gram-positive and Gram-negative food pathogens. The lytic activity of LysZC1 on Gram-positive bacteria showed a remarkable OD_600_ reduction in the case of *B. cereus* as compared to *S. aureus* (Figure 5A). Our results show the synergistic effect of 100 μg/mL LysZC1 in presence of 3 mM EDTA against Gram-negative bacteria (Figure 5B). Upon treatment with LysZC1 and EDTA, the OD_600_ of bacterial suspension decreased significantly, as shown in Table 1. The data thus show that LysZC1 demonstrates the highest activity against *B. cereus* and hence we carried out our further experiments using it as the target organism.

### 3.5. LysZC1 Efficiently Kills Bacillus cereus at Logarithmic and Stationary Phase

**Figure 6:**
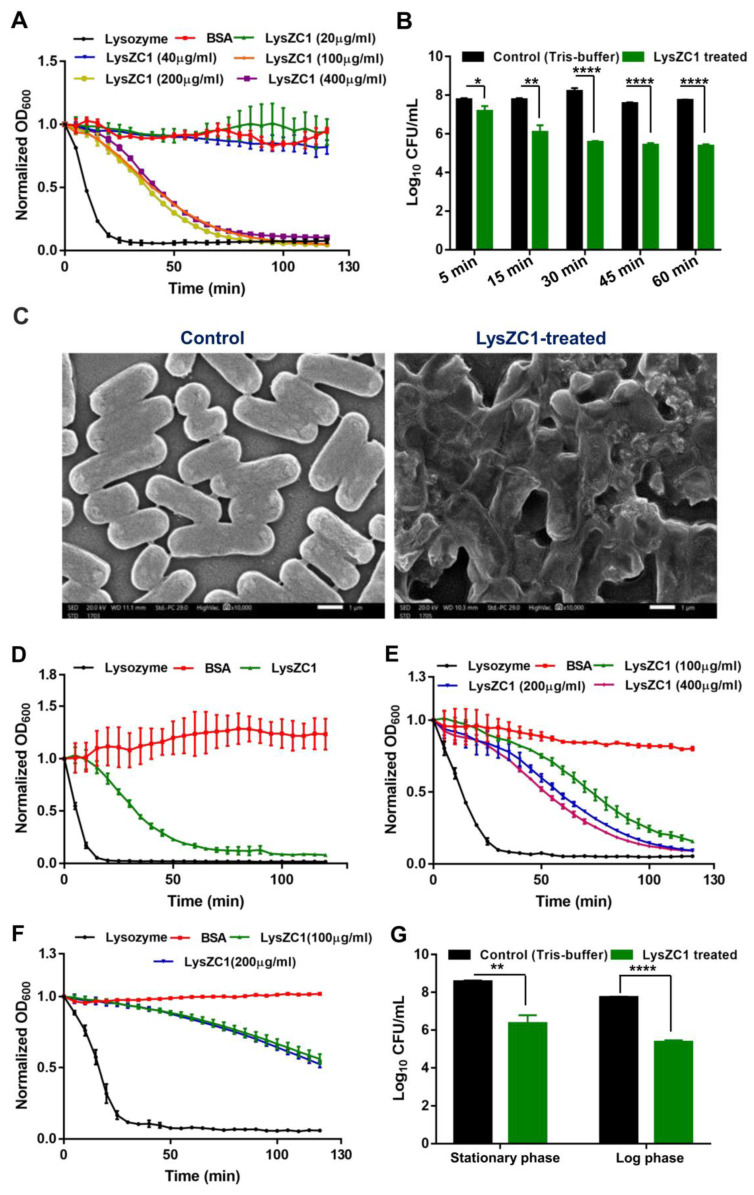
The lytic activity of LysZC1 on *Bacillus cereus*. (**A**) Estimation of Minimum Inhibitory Concentration of LysZC1 against logarithmic phase of *B. cereus*. (**B**) CFU reduction assay of LysZC1 against Logarithmic phase of *B. cereus.* Data is shown as Mean ± SD. The data is statistically significant considering *p* ≤ 0.05 (*), *p* ≤ 0.01 (**), and *p ≤* 0.0001 (****); (**C**) SEM micrograph of untreated (left panel) and LysZC1-treated (right panel) *B. cereus* strains; (Assessment of the antibacterial activity of LysZC1 against *B. cereus* at both logarithmic and stationary phases by turbidity reduction assay. The line graphs represent the activity of LysZC1 against *B. cereus* with OD_600_ = 1 (**D**), OD_600_ = 2 (**E**), and OD_600_ = 3 (**F**). (**G**) CFU reduction of logarithmic and stationary phase *B. cereus*. Bacteria at both phases were treated individually with 100 μg/mL of LysZC1 for 1 h at 37 °C. Data are shown as Mean ± SD.

In order to develop LysZC1 as a potential molecule to kill *B. cereus*, we first determined the minimum inhibitory concentration of LysZC1 against *Bacillus cereus* by treating the bacterium with different concentrations of the enzyme and monitoring the reduction in OD_600_. Figure 6A shows that the MIC of LysZC1 is 100 μg/mL. We further examined the bactericidal activity of LysZC1 against the log-phase *B. cereus* cells by measuring the survival of bacterial cells in a time-killing experiment. Our data showed that 100 μg/mL LysZC1 is able to kill log-phase *B. cereus*, and resulted in more than 2 log_10_ reduction in the bacterial count (CFU/mL) after incubation for 1 h (Figure 6B) with a lytic activity of 40.7 ± 1.5%. These results clearly showed that LysZC1 has robust bactericidal activity, which suggests a potential use of LysZC1 as an antibacterial agent.

To visualize the killing of *B. cereus* by LysZC1, we examined the morphology of *B. cereus* after treatment with LysZC1 using scanning electron microscopy. Our SEM data clearly shows severely damaged and disrupted *B. cereus* cells upon exposure to 100 μg/mL of LysZC1 for 2 h, whereas the untreated cells show normal cellular morphology (Figure 6C), which further confirms the killing activity of LysZC1 on *B. cereus*.

We next examined if the log and stationary phases of *B. cereus* growth affect the activity of LysZC1 by treating the bacteria in those growth phases with the LysZC1 protein before enumerating the viable cells. Our turbidity reduction data shows a significant decline with log-phase culture (OD_600_ = 1 and 2; Figure 6D,E). Figure 6F shows that although the decrease in OD_600_ is less as compared to the log-phase, but it is significant enough to remove the bacteria if treated for a longer period. Figure 6G gives a depiction of the above data in terms of the count of viable cells, which suggests a significant (*p* ≤ 0.05) reduction of viable bacterial cells even for the cells taken from the stationary phase. We thus conclude that LysZC1 is able to kill both the rapidly growing (log- phase) and the stationary-phase *B. cereus* cells efficiently.

### 3.6. LysZC1 Demonstrates Lytic Activity in a Wide Range of Temperature, pH and Prolonged Storage

**Figure 7:**
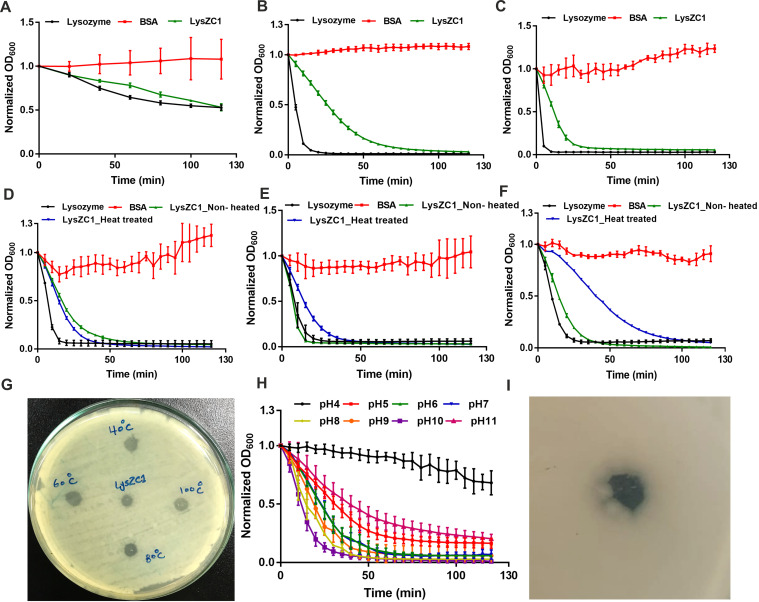
LysZC1 is active at different temperatures and after prolonged storage. A-F show the bactericidal activity of LysZC1 against *B. cereus* after incubating the enzyme at different temperatures ((**A**)—10 °C, (**B**)—25 °C, (**C**)—45 °C, (**D**)—60 °C, (**E**)—80 °C, and (**F**)—100 °C). Lysozyme and BSA were used as positive and negative controls and the data was plotted as Mean ± SD. In panels (**D**–**F**), LysZC1 was first incubated at the indicated temperature for 10 min, followed by cooling to room temperature, before proceeding for activity assay. Panel (**G**) shows the same effect on a TSA plate covered with *Bacillus* lawn, treated with LysZC1 after heating at different temperatures as indicated. Panel (**H**) depicts the effect of different pH on the activity of LysZC1. Panel (**I**) shows the same plate assay but after storing the enzyme at 4 °C for 11 months. The zone of clearance in both the panels represents bacterial killing.

We next carried out activity assays at different temperatures ranging from 10 °C to 100 °C. LysZC1 is able to show activity at temperatures as low as 10 °C (Figure 7A). The thermal stability data suggests that LysZC1 at 100 μg/mL shows phenomenal lytic activity at 25, 45, 60, 80, and 100 °C (Figure 7B–F). In panels Figure 7D–F, however, the protein was first heated at these temperatures, cooled to room temperature, and then the activity of this protein was assayed in the turbidity reduction assay with *B. cereus* as substrate. Nevertheless, our data show LysZC1 is able to tolerate a broad range of temperatures and retains activity even after incubation at temperatures as high as 100 °C. Figure 7G represents the same lytic effect of LysZC1 on the *Bacillus cereus* lawn. It indicates that LysZC1 is a highly thermo-stable protein. Besides temperature, pH is also an important factor for the growth of bacteria, and the activity of any enzyme. To address the effect of pH on the activity of LysZC1, we prepared the substrate with buffers of different pH and treated it with 100 μg/mL. We found out that LysZC1 demonstrates exceptional activity over a wide range of pH (Figure 7H). Although LysZC1 appears to be slightly alkalophilic in nature, showing maximum activity within pH 8–10. We next asked if LysZC1 can indeed be stored long term without losing activity. Our data show that the protein is able to form a zone of clearance on *B. cereus* bacterial lawn (Figure 7I) even after being stored for 11 months at 4 °C.

The results indicated that LysZC1 has the highest bactericidal activity against *B. cereus*, when administered at a wide range of temperatures and pH, can be stored for a long duration, and has the potential to be used as an antibacterial agent, although the data from the in-vivo experiments are currently lacking.

## 4. Discussion

Despite significant advances in food sanitation procedures and pathogen tracking, foodborne diseases remain among the leading causes of hospitalization and death globally [40]. *B. cereus* is one of the most common food-poisoning pathogens worldwide. It releases enterotoxins that contribute to its pathogenicity, as well as a preformed cereulide toxin that causes disease once the poisoned food is consumed [41,42]. To eliminate microbial infections, decontamination of the livestock is generally achieved by the addition of antibiotics to their feed. The equipment and farms are sanitized by disinfectants and biocides. This is called primary production which is followed by the handling and processing of the raw material in food industries, which includes the use of antibiotics, sanitizers, and preservatives in the final product. This food chain ends with the consumer of the food who also indirectly intakes the antimicrobials along with the food. This is also called farm to fork continuum [18,43]. Such regular use of these antimicrobials at their sub-lethal and lethal doses has led to the development of antimicrobial resistance in these pathogens. For instance, *Pseudomonas aeruginosa* is resistant to polymyxin B, tetracycline, and ciprofloxacin [44]; *E. coli* is resistant to triclosan [45], *Klebsiella pneumoniae* is resistant to colistin [46], *Salmonella* spp. is resistant to nalidixic acid [47], and *Bacillus* spp. and *Enterococcus* spp. from organic foods are resistant to ampicillin, cefotaxime, and sulfamethoxazole [48]. This has led to an increasing microbial count in different stages of the food chain and calls for effective alternative ways to bacterial management [43].

Phage encoded lysozymes are considered a potential alternative antibacterial tool for combating the antimicrobial resistance property of pathogens. These enzymes either target the peptidoglycan post-adsorption (Tail lysozymes) or during the lysis of the bacteria (endolysins) for phage progeny release, and have been previously used as potential biocidal agents in food materials. For example, anti- *Listeria* endolysin Ply511 (peptidoglycan hydrolase) gene was expressed in *Lactococcus* spp. in fermented products for using them as bio-preservatives against *Listeria monocytogenes* and other foodborne pathogens [49]. Other examples include the reduction of *Salmonella* spp. in chickens by oral administration of purified truncated *Salmonella* phage tail spike endoglycosidase [50], and the use of phage endolysin CHAP8H3b to remove *S. aureus* in raw milk [51]. We present here the structural and functional validation of a putative lysozyme LysZC1 isolated from the newly discovered lytic *Pseudomonas* phage ZCPS1. We here show that this molecule has the potential in fighting the most infectious foodborne pathogen *Bacillus cereus*, laying the groundwork for future research to engineer and exploit LysZC1 to control MDR pathogens found in food products.

Generally, lysins from bacteriophages infecting Gram-negative bacteria are small single-domain globular structures with the enzymatically active or catalytic domain (EAD) and without the cell wall binding domain (CBD) [52]. Recent studies also indicate the existence of Gram-negative phage endolysins with a modular structure, one or two CBDs at the N-terminal, whereas the EAD module is located at the C-terminal [52]. Our annotation data shows that ZCPS1 phage comprises ORF114, which encodes a 165-amino acid protein with a deduced molecular weight of ~18.8 kDa and is predicted to function as lysozyme with muramidase activity, as well as a gene encodes for a holin and other structural proteins. For other lysins, there is no additional coding gene (Figure S3). Our structural alignment data with few available phage proteins and multiple sequence alignment data (Figure S1) of LysZC1 shows that LysZC1 is a phage-encoded lysozyme with glycosidase hydrolase activity.

LysZC1 shows sequence similarity with few phage baseplate or tail lysozymes, which indicates that it belongs to the muramidase group, and is responsible for cleaving of β-1,4 glycosidic bond between *N*-acetylmuramic acid (MurNAc) and *N*-acetyl-D-glucosamine (GlcNAc) residues, a structural feature of the peptidoglycan cell wall present in both Gram-positive and Gram-negative organisms. This suggests that the LysZC1 is a broad-spectrum lysin encoded by *Pseudomonas* phage ZCPS1. The tail lysozymes have also been engineered to enhance their muralytic activity. For instance, broad-spectrum lysozyme from staphylococcal phage K showed significant activity against drug-resistant *S. aureus* [53]. The more effective chimeric enzyme P128 was prepared by joining the catalytic hydrolase domain of K-encoded lysozyme and the cell wall binding domain of a bacteriocin called lysostaphin. This was tested and verified to be effective against methicillin-resistant *S. aureus* strains [54].

Our zymography data with *Micrococcus lysodeikticus* which is a Gram-positive bacterium, confirms the lytic property of recombinant LysZC1. We also tested the activity of LysZC1 against commonly known Gram-positive and Gram-negative foodborne pathogens viz. *B. cereus* ATCC 11778 and MTCC 1306, *Staphylococcus aureus* MTCC 1430T, *Salmonella typhimurium* ATCC 14028, *Pseudomonas aeruginosa*, *E. coli* ATCC 8739, *Shigella sonnei* ATCC 29930. *S. aureus* and few species of *Micrococcus* cause curing, salting and fermentation of meat products [54,55]. *P. aeruginosa* causes proteolysis and hydrolytic rancidity of raw milk and dairy products. It is also responsible for pigmentation, odor, and slime production on the surface of cheese [55,56]. *Salmonella sp.* usually dwells in the intestines of livestock and its outbreaks are mostly associated with eggs, raw milk, and poultry [56]. The outbreaks associated with pathogenic *E. coli* were found in raw fruits and vegetables [56]. *S. sonnei* is the most prevalent pathogen among *Shigella sp.,* mostly associated with the outbreak of milk, salads, chicken, etc., and *K. pneumoniae* causes food poisoning from chicken and meat. Our turbidity reduction assays against these Gram-negative bacteria clearly demonstrate that LysZC1 in synergism with EDTA shows maximum cell lysis activity. EDTA disrupts the charge distribution in LPS by removing the charge screening effect of the divalent cations, which leads to the electrostatic repulsion between adjacent LPS molecules [57], and thus allows the enzyme to access peptidoglycan. Among the five pathogens tested, while LysZC1 was found to be most active against *Pseudomonas aeruginosa*, it indeed showed significant activity against other bacteria. Our data against Gram-positive organisms exhibit the exceptional activity of LysZC1 against *B. cereus* and nearly no activity against *S. aureus*. Our data thus suggests that LysZC1 can be used to decontaminate surfaces or food products that are contaminated with multiple organisms. As LysZC1 is a *Pseudomonas* phage-encoded enzyme demonstrating the highest activity against *B. cereus*, it also overcomes the probability of the emergence of phage-resistant properties because of the specific phage-host interactions. 

Our study also demonstrates that endolysin LysZC1 exhibits a lytic effect on the *B. cereus* bacterial strain; here, the antibacterial activity of LysZC1 was confirmed by its ability to reduce the viability of *B. cereus* bacterial cells. Different studies have shown that endolysins are more active against logarithmic phase bacteria than those from the stationary phase [37]. In addition, another study indicated that bacterial phases have an insignificant effect on lysin activity [58]. Consequently, LysZC1 activity was also monitored against logarithmic and stationary phase *B. cereus* cells for 1 h at 37 °C. Our data showed that LysZC1 has almost indifferent activity on both logarithmic and stationary phase bacteria. SEM analysis results further showed that *B. cereus* cells could be lysed upon treatment with LysZC1, which indicates that LysZC1 has robust bactericidal activity against *B. cereus* bacteria. This robust activity of a *Pseudomonas* phage lysozyme indicates that LysZC1 employs a lysozyme-like domain showing broad-spectrum activity. This might be due to targeting the similarity in structure of Gram-positive and Gram-negative bacteria in terms of the presence of N-acetyl glucosamine and N-acetyl muramic acid.

Apart from the nutritional and physical characteristics of food, the fate of micro-organisms also depends on several extrinsic and intrinsic factors such as temperature, pH, water activity, and redox potential. These factors are important as they can be conveniently manipulated to remove microbial load during food processing.

A recent study has demonstrated that endolysin’s stability is crucial to its utility and application [59]. When subjected to thermo-stability tests, some endolysins retain their activity even after prolonged heating. For endolysin from phages infecting Gram-negative bacteria, *Pseudomonas* phage endolysins KZ144 and EL188 activity remained high after 10 min at 50 °C [52]. On the other hand, the T4 phage lysozyme retained only a small yet significant portion of its activity after only 5 min at 65 °C [60]. Furthermore, a study by Walmagh et al. showed that endolysin PVP-SE1gp36 remains thermo-resistant up to 90 °C [61]. In contrast, the phage endolysin Lys68 activity was completely lost upon exposure to 100 °C for 30 min but the enzyme remains active after 30 min incubation at 40 °C and 60 °C; however, it did maintain 54.7% of its residual activity at 80 °C [62]. It is important to mention the endolysins encoded by *Thermus scotoductus* phages Ph2119 and Ts2631. The Tsc2631 endolysin retained 65% of its activity after 2 h at 95 °C (Tm = 99.8 °C), while Ph2119 endolysin was active at 90% after 6 h of incubation at 95 °C [63]. The endolysin LysB4 from *Bacillus cereus* infecting bacteriophage B4 shows alkalophilic and thermophilic properties [64]. In this study, we have examined the effect of temperature and pH on the activity of LysZC1. To test the thermo-stability of LysZC1, we checked the activity of LysZC1 against *Bacillus cereus* by maintaining the temperature at 10, 25, and 45 °C and also after heating the enzyme at 60, 80, and 100 °C followed by cooling to room temperature and assaying its enzyme activity. Our results showed that LysZC1 remains active in a wide range of temperatures, and can sustain its activity after heating at high temperatures for 10 min. We believe that this will be of great advantage as LysZC1 can now be used in food processes requiring lower, intermediate, and high-temperature incubation. We also carried out the enzyme activity at different pH. Although *Bacillus cereus* has been reported to show growth in the range of pH 4.9–9.5, its optimum growth occurs at pH 6 and 7 [65]. Nevertheless, here, we widened our assay pH range from 4–11 to examine the effect of pH on the activity of the enzyme. A highly acidic environment (pH 2) was found to kill *Bacillus cereus* cells and also hampered LysZC1 activity, whereas a mildly acidic (pH 4) and highly basic (pH 11) environment although did not affect the growth of *Bacillus* significantly, retarded the enzyme’s activity. The data suggest that the enzyme efficiently works in the range of pH 5–10 and shows optimum activity towards alkaline pH from 8–10 at 37 °C. We have additionally observed extraordinary stability of LysZC1 even after prolonged storage at 4 °C, which immediately qualifies it to be used as a bio-preservative. 

## 5. Conclusions

Our data presented here allow us to conclude that the LysZC1 shows the highest activity towards *B. cereus* (~90%) among different food pathogens tested. LysZC1 is antibacterial against the log as well as stationary phase *B. cereus* culture. Apart from *Bacillus cereus*, LysZC1 shows ~50% bactericidal activity against *Pseudomonas aeruginosa*. LysZC1 is a thermo-stable endolysin exhibiting significant activity after prolonged storage and heat treatment and acidic, neutral, and basic conditions. Thus, we claim that LysZC1 is a potential antibacterial and preserving agent against *Bacillus* contamination in food industries.

## Figures and Tables

**Table 1:** Antibacterial effect of LysZC1 against various Gram-positive and negative food pathogens.

Pathogen Name	Gram Character	Percentage OD_600_ Reduction
*Bacillus cereus*	Gram-positive	87.3%
*Staphylococcus aureus*	Gram-positive	18.33%
*E. coli*	Gram-negative	38.31%
*Salmonella typhimurium*	Gram-negative	25.5%
*Shigella sonnei*	Gram-negative	26%
*Pseudomonas aeruginosa*	Gram-negative	52.3%
*Klebsiella pneumoniae*	Gram-negative	18.6%

## Data Availability

The sequence of *Pseudomonas* phage ZCPS1 Endolysin was deposited in GenBank under accession No. UPO63074.

## Supplementary Materials

The following supporting information can be downloaded at: https://www.mdpi.com/article/10.3390/v15020520/s1, Figure S1: Multiple sequence alignment of LysZC1 with various phage lysin proteins showing high sequence similarity; Figure S2: Constraint based multiple sequence alignment of LysZC1 with proteins showing sequence similarity and used for structural alignment; Figure S3: Schematic representation of the annotated genes of the structural module of ZCPS1phage; Table S1: List of phage lysin proteins showing high sequence similarity with amino acid sequence of LysZC1 protein; Table S2: List of templates with maximum similarity used for structural alignment with predicted LysZC1 model.
