# Supplementary material for: Pseudomonas Phage ZCPS1 Endolysin as a Potential Therapeutic Agent
Source: Viruses. 2023 Feb 13;15(2):520. doi: 10.3390/v15020520 (PMC9961711; doi:10.3390/v15020520)
Supplement: Supplementary file 1 [file viruses-15-00520-s001.zip › viruses-1975450-supplementary.pdf]

Supplementary Tables:

Table S1: List of phage lysin proteins showing high sequence similarity with amino acid sequence of LysZC1 protein.

Alignment no.	Description	Identity
Query_10001	LysZC1	--
Query_10002	WP_015973755.1:1-165 glycoside hydrolase family protein [*Klebsiella pneumoniae*]	99.39 %
Query_10003	YP_007183210.1:1-165 glycoside hydrolase family protein [*Pseudomonas* phage vB_PaeP_p2-10_Or1]	98.79%
Query_10004	QIQ64397.1:1-165 lysozyme [*Pseudomonas* phage Epa1]	98.37%
Query_10005	ATW62317.1:1-165 lysozyme [*Pseudomonas* phage Delta]	97.58%
Query_10006	YP_009007856.1:1-165 glycoside hydrolase family protein [*Pseudomonas* phage TL]	84.85%
Query_10007	QSH71737.1:1-123 lysozyme [*Pseudomonas* phage vB_PaeP_fHoPae04]	86.73%
Query_10008	YP_008857868.1:1-132 lysozyme [*Pseudomonas* phage phiIBB-PAA2]	86.36%
Query_10009	YP_001671940.1:1-132 T4-like lysozyme [*Bruynoghe* virus LUZ24]	85.61%
Query_10010	YP_009209320.1:1-132 baseplate hub protein [*Pseudomonas* phage DL54]	85.61%
Query_10011	QVJ13268.1:1-132 baseplate hub [*Pseudomonas* phage PSA31]	84.85%
Query_10012	YP_006382529.1:1-166 lysozyme [*Pseudomonas* phage tf]	63.86%
Query_10013	URG14323.1:1-113 lysozyme [*Pseudomona*s phage vB_PaeP_PaCe]	86.73%
Query_10014	AYJ73846.1:1-113 baseplate hub protein [*Pseudomonas* phage SaPL]	86.73%
Query_10015	QDH46231.1:1-113 baseplate hub [*Pseudomonas* virus Pa223]	86.73%
Query_10016	QDH46160.1:1-113 baseplate hub [*Pseudomonas* virus Pa222]	86.73%
Query_10017	AWY03080.1:1-132 lysozyme [*Pseudomonas* phage SCYZ1]	63.64%
Query_10018	YP_006907044.2:1-162 lysozyme [*Pseudomonas* phage UFV-P2]	57.41%
Query_10019	UMO76430.1:1-128 lysozyme [*Pseudomonas* phage Broder Salsa]	59.38%
Query_10020	YP_009622530.1:1-136 lysozyme [*Pseudomonas* phage Bjorn]	56.62%
Query_10021	KAB2362637.1:1-81 lysozyme, partial [*Escherichia coli*]	82.72%

Table S2: List of templates with maximum similarity used for structural alignment with predicted LysZC1 model

S. No.	Template protein structure	PDB ID	RMSD	TM score	Sequence Identity	Equivalent Residues	Reference Coverage	Target Coverage
1	Lysozyme 056 from Deep neural language modeling	7RGR	3.03	0.67	27.63%	140	85%	84%
2	Crystal structure of a pesticin and T4-lysozyme chimera	4ARJ	3.18	0.38	27.68%	144	87%	45%
3	Muramidase domain of SpmX from *Asticaccaulis excentricus*	6H9D	2.52	0.73	34.52%	140	85%	95%
4	Endolysin from *Escherichia coli* O157:H7 phage FAHEc1	7M5I	1.28	0.78	38.64%	138	84%	86%
5	Crystal structure of muramidase from *Acinetobacter baumannii* AB 5075UW prophage	6ET6	1.36	0.79	27.08%	140	85%	94%

Supplementary Figures:

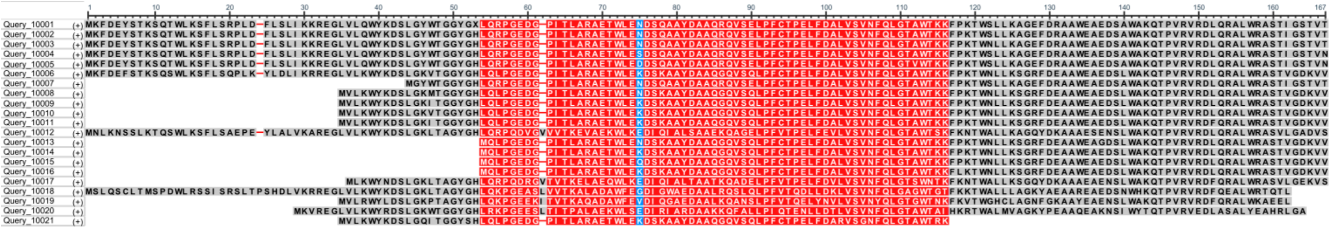

Figure S1: Multiple sequence alignment of LysZC1 with various phage lysin proteins showing high sequence similarity. The highly and partially conserved residues are highlighted with red and blue colors, respectively.

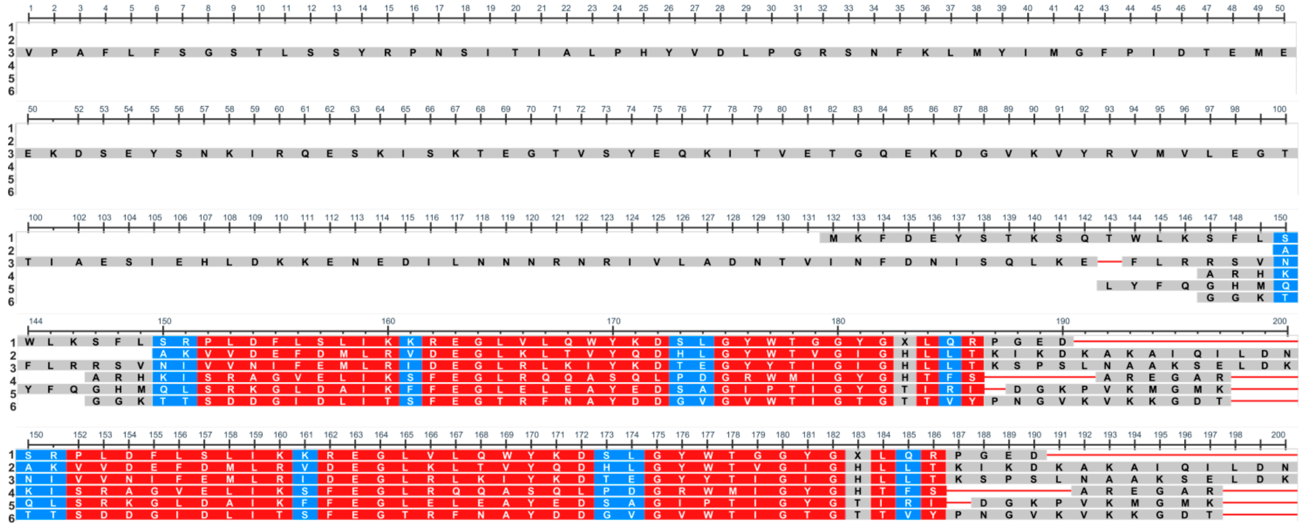

Figure S2: Constraint based multiple sequence alignment of LysZC1 with proteins showing sequence similarity and used for structural alignment. The highly and partially conserved residues are highlighted with red and blue colors, respectively. Lane 1 represents LysZC1 protein while lanes 2, 3, 4, 5, and 6 represent LysZC1 protein alignment with Lysozyme 056 from deep neural language modeling, T4-lysozyme, Muramidase domain of SpmX from *Asticaccaulis excentricus*, Endolysin from *E. coli* O157:H7 phage FAHEc1, and Muramidase from *Acinetobacter baumannii* AB 5075UW prophage, respectively.

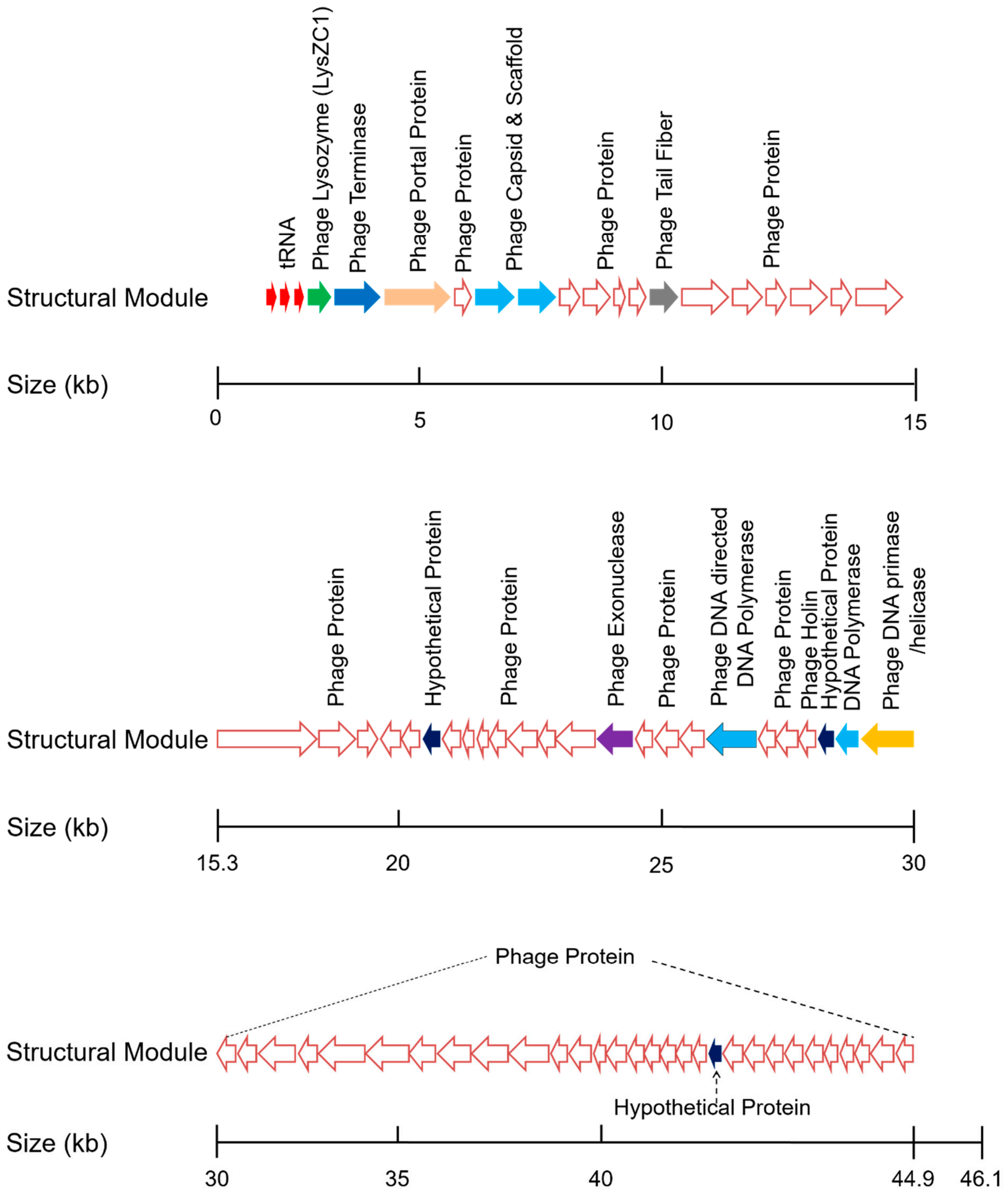

Figure S3: Schematic representation of the annotated genes of the structural module of ZCPS1 phage.
